# *Trp53* Deficiency Increases the Frequency of *Hprt* Mutant T Lymphocytes Following High‐LET Iron Particle Irradiation of Mice

**DOI:** 10.1002/em.70084

**Published:** 2026-10-07

**Authors:** Vernon E. Walker, Dale M. Walker, Vasily N. Dobrovolsky, Louise Lutze‐Mann

**Affiliations:** ^1^ New York State Department of Health Wadsworth Center Albany New York USA; ^2^ Department of Pathology and Laboratory Medicine, Larner College of Medicine University of Vermont Burlington Vermont USA; ^3^ Experimental Pathology Laboratories Sterling Virginia USA; ^4^ The Burlington HC Research Group Inc. Jericho Vermont USA; ^5^ Division of Genetic and Molecular Toxicology U.S. Food and Drug Administration, National Center for Toxicological Research Jefferson Arkansas USA; ^6^ School of Biotechnology and Biomolecular Sciences University of New South Wales Sydney Australia

## Abstract

Charged particles like iron‐56 ions impose health risks in long‐term manned space exploration. This study was designed to define the impact of *Trp53* status upon the manifestation of mutations in the *hypoxanthine‐guanine phosphoribosyltransferase* (*Hprt*) gene in splenic T‐cells following exposure of *Trp53* wild‐type, hemizygous, and nullizygous *lacZ* transgenic mice to 1 Gy ^56^Fe particles. In male and female mice necropsied at 2, 4, and 8 weeks postexposure, *Hprt* mutant frequencies in splenic T‐cells (measured via the *Hprt* mutation cloning assay) increased significantly over time to peak at 4 weeks postexposure before decreasing to lower but still elevated levels. Mutagenic potency estimates based upon the change in mutant frequency over time in exposed versus respective control groups showed that the total mutagenic response depended upon the *Trp53* genetic status, with the mutagenic burden of 1 Gy being threefold and 5.3‐fold greater in *Trp53* ^+/−^ and *Trp53* ^−/−^ mice, respectively, compared to *Trp53* ^+/+^ mice.

## 1 Introduction

Cancer risk from exposure to galactic cosmic rays is a long‐term concern associated with prolonged space travel (Chang et al. 2000; Turker et al. 2017; Katsube et al. 2022). Charged particles with high linear energy transfer (LET) present in galactic cosmic rays have a greater potential for causing complex DNA damage/mutations than low‐LET photon radiations such as γ‐ or x‐rays. While high atomic number and energy (HZE) charged particles constitute only 0.9% of galactic cosmic rays, they comprise ~88% of the estimated ionizing dose equivalent from cosmic rays (Katsube et al. 2022). Iron particles are the most densely ionizing HZE particles, occurring in relatively large amounts compared to other heavy nuclei in space.

The status of the *TP53* tumor suppressor gene in humans (*Trp53* in mice) is a major factor influencing the response to radiation‐induced DNA damage (Chang et al. 2000; Chang et al. 2001a, 2001b; Fernandez et al. 2008). The p53 protein in humans (Trp53 in mice) binds to specific DNA response elements and induces the expression of genes with roles in cell cycle arrest, DNA repair, apoptosis, senescence, autophagy, ferroptosis, and/or metabolism (Duffy et al. 2022; Blackburn and Jerry 2002). In adult mice, radiosensitivity to tumorigenesis is dependent upon *Trp53* genetic status, with pre‐ and post‐irradiation expression of Trp53 protein target genes, which are involved in cell cycle arrest and apoptosis that vary between tissues (Chang et al. 2001a, 2001b). Radiation‐induced DNA damage in humans may pose a greater risk for tissue‐specific mutagenesis and long‐term health consequences in p53‐deficient cells than in cells with wild‐type p53.

T lymphocytes are a relevant bioindicator because they are highly radiosensitive and radiation exposure is a risk factor for acute lymphocytic leukemia (Grygoryev et al. 2016). Several studies have evaluated the mutagenicity of high‐LET radiation in splenic T‐cells using mice with integrated transgenes or the endogenous autosomal *adenine phosphoribosyltransferase* (*Aprt*) reporter gene to screen for mutations (Chang et al. 2001a; Yatagai et al. 2001; Masumura et al. 2002; Chang et al. 2007; Grygoryev et al. 2016). The studies reported herein were conducted to determine the relative mutagenic potency of an acute dose of 1 Gy ^56^Fe particles in splenic T‐cells of *lacZ* mice with different *Trp53* genetic statuses, by measuring the manifestation or change in the frequency of mutations over time (Meng et al. 1998; Walker et al. 1999) in the endogenous X‐linked *hypoxanthine‐guanine phosphoribosyltransferase* (*Hprt*) reporter gene.

## 2 Materials and Methods

### 2.1 Animals, Husbandry, and Exposures

C57Bl/6‐TgN(*LacZ*pl)60 transgenic mice were bred with *Trp53* knockout mice (Jackson Laboratory, Bar Harbor, ME) to obtain hybrids that were bred to produce further generations of mice that were *Trp53* wild‐type (*Trp53* ^+/+^), *Trp53* hemizygous (*Trp53* ^+/−^), or nullizygous (*Trp53* ^−/−^) (Chang et al. 2000; Chang et al. 2001a, 2001b). *LacZ* transgenic mice, separated by sex and *Trp53* status, were housed in conformance with National Research Council guidelines in temperature‐ and humidity‐controlled rooms with a 12‐h light/dark cycle. Animals were free of virus titers, as determined by standard mouse antibody assays. All procedures involving animals were approved by the Institutional Animal Care and Use Committees where animal experiments were performed. Groups of female and male *lacZ* mice of differing *Trp* status were sent to Brookhaven National Laboratory, and after acclimation, animals were exposed acutely to 0 or 1 Gy of 1 GeV/amu iron (^56^Fe) particle radiation at the Alternating Gradient Synchrotron (Chang et al. 2000; Chang et al. 2001a, 2001b). One set of mice (12 weeks old at irradiation) was sent overnight to the Wadsworth Center to evaluate the frequency of *Hprt* mutant T‐cells (MF) isolated from spleen at 8 weeks postexposure (Experiment 1). Subsequent sets of mice (10–12 weeks old at irradiation) were used to measure *Hprt* MFs at 4 and 8 weeks postexposure (Experiment 2) and 2 weeks postexposure (Experiment 3).

### 2.2 Culture of *Hprt* Mutant Lymphocytes

Cell culture reagents were purchased from indicated sources: 6‐thioguanine and β‐mercaptoethanol, Sigma‐Aldrich (St Louis, MO); concanavalin A, Worthington Biochemical (Freehold, NY); Lympholyte M, Accurate Chemical and Scientific Corp. (Westbury, NY); HEPES buffer, Fisher Scientific (New York, NY); HL‐1, BioWhittaker (Walkersville, MD); and rat T‐STIM supplement and mouse recombinant interleukin‐2 (IL‐2), Collaborative Biomedical Products (Bedford, MA). Other medium components, including RMPI 1640 medium, fetal bovine serum, l‐glutamine, nonessential amino acids, penicillin–streptomycin, and sodium pyruvate, were obtained from Media and Glassware, Support Service Group in the Wadsworth Center (Albany, NY). A current alternate source for critical reagents is noted elsewhere (Judice et al. 2023).

Mice were euthanized by CO_2_ asphyxiation and cervical dislocation; spleens were removed aseptically for isolation of lymphocytes, and *Hprt* MFs were measured in blinded fashion based on coded ear tag numbers. Primary splenic T‐cells were stimulated and cultured in the presence of IL‐2, concanavalin A, and 6‐thioguanine to select for *Hprt* mutant clones or cultured with irradiated feeder cells without 6‐thioguanine to determine T‐cell cloning efficiency, as previously described (Meng et al. 1998; Walker et al. 1999; Judice et al. 2023).

### 2.3 Statistical Analyse*s*

The Mann–Whitney *U*‐statistic was used to describe the differences in cloning efficiency and MF values between sham‐exposed control and radiation treatment groups of female and male mice combined for each distinct *Trp53* genotype at specific time points post‐exposure. As a separate evaluation, *p* values for comparing differences in induced radiation effects in groups of mice based upon *Trp53* status (at specific time points postexposure) were generated using a two‐way ANOVA and Tukey's post hoc test for multiple pairwise comparisons, with radiation exposure or no exposure as one independent variable and *Trp53* genotype (*Trp53* ^+/+^, *Trp53* ^+/−^, or *Trp53* ^−/−^) as the second independent variable. Due to small sample size, data from both female and male mice in the same control or treatment group were generally combined for statistical analysis. All statistical analyses were performed using SigmaStat software (version 3.01; Systat Software Inc., San Jose, CA). *p* < 0.05 was considered significant. A novel mathematical approach previously developed to compare the mutagenic potency of genotoxic chemicals in rodents exposed at similar or different ages (Meng et al. 1998; Walker et al. 1999; Judice et al. 2023) was used to derive an estimate of mutagenic potency by plotting the *Hprt* MFs for individual exposed and control animals necropsied at differing time points postexposure and then determining the difference in the areas under the resulting mutant T‐cell manifestation curves for irradiated versus control animals of differing *Trp53* status. Briefly, this objective was accomplished by fitting a non‐linear model to the MF data, integrating areas under the mutant manifestation curves for exposed versus control mice, and computing 95% confidence intervals.

## 3 Results

*Trp53* genetic status in mice had no apparent effects on the cloning efficiencies and spontaneous *Hprt* MFs in splenic T‐cells. The average cloning efficiency in sham‐exposed control female and male mice was 6.2% (range of 2.0%–12.4%), with no significant differences based upon *Trp53* status in 17, 16, and 16 *Trp53* ^+/+^, *Trp53* ^+/−^, *Trp53* ^−/−^ mice, respectively. In irradiated female and male mice, the cloning efficiencies averaged 5.6% (range of 2.2%–11.4%) in 20, 16, and 15 *Trp53* ^+/+^, *Trp53* ^+/−^, and *Trp53* ^−/−^ mice, respectively. Finally, the average background *Hprt* MF in female and male control mice was 1.4 ± 0.6 × 10^−6^ (*n* = 50), with no significant differences found based upon *Trp53* status. These cloning efficiency and *Hprt* MF values resembled those reported previously for splenic T‐cells of control mice (reviewed in Judice et al. 2023).

A pilot study was performed to assess the mutagenic effects in T‐cells isolated from spleens at 8 weeks after acute irradiation of female and male *lacZ* mice with different *Trp53* status at 12 weeks‐of‐age (Table 1). Exposure to 1 Gy ^56^Fe particles led to significant increases in *Hprt* MFs of 2.0 ± 0.4 × 10^−6^, 9.1 ± 1.8 × 10^−6^, and 14.7 ± 1.8 × 10^−6^ in *Trp53* ^+/+^, *Trp53* ^+/−^, and *Trp53* ^−/−^ mice, respectively, which were 1.8‐fold, 6.1‐, and 11.3‐fold greater than the background MFs in respective control groups of female and male mice combined (respective *p*‐ values = 0.025, 0.00019, and 2.6 × 10^−6^). *Hprt* MFs in irradiated *Trp53* ^−/−^ mice were significantly greater than in exposed *Trp53* ^+/+^ (*p* = 1.4 × 10^−6^) and *Trp53* ^+/−^ mice (*p* = 0.004). Also, MFs in irradiated *Trp53* ^+/−^ mice were significantly elevated compared to exposed *Trp53* ^+/+^ mice (*p* = 0.0002).

**TABLE 1:** *Hprt* mutant frequencies in T lymphocytes from spleens of female and male *lacZ* transgenic mice, with different *Trp53* genetic status, at 8 weeks after acute iron particle irradiation^a^.

Dose (Gy)	Code number	*Trp53* status	Sex	Observed mutant frequency (×10^−6^)
0	N8C1	+/+	F	1.31
	N8C2		F	1.11
	N8C3		F	0.95
	N8C4		M	0.91
	N8C5		M	1.42
	N8C6		M	0.98
				*x* = 1.10 ± 0.2 (SD)
1	N9.1	+/+	F	1.19
	N9.2		F	1.63
	N9.3		F	1.38
	N9.4		M	2.41
	N9.5		M	3.60
				*x* = 2.04 ± 0.4 *p* = 0.025, irradiated vs. control +/+ mice^b^
0	HTC1	+/−	F	0.80
	HTC2		F	1.38
	HTC3		M	1.96
	HTC4		M	1.71
				*x* = 1.46 ± 0.5
1	HT2.1	+/−	F	7.55
	HT2.2		F	11.04
	HT2.3		M	8.79
	HT2.4		M	Dead on arrival
				*x* = 9.1 ± 1.8 *p* = 0.00019, irradiated vs. control +/− mice^b^ *p* = 0.0002, induced effect in +/− vs. +/+ mice^c^
0	HMC13	−/−	F	1.43
	HMC14		F	1.28
	HMC15		M	1.11
	HMC16		M	1.32
				*x* = 1.3 ± 1.3
1	HM7.1	−/−	F	16.2
	HM7.2		F	12.2
	HM7.3		M	14.8
	HM7.4		M	15.6
				*x* = 14.7 ± 1.8 *p* = 2.6 × 10^−6^, irradiated vs. control −/− mice^b^ *p* = 1.4 × 10^−6^, induced effect in −/− vs. +/+ mice^c^ *p* = 0.004, induced effect in −/− vs. +/− mice^c^

^a^ C57Bl/6 *lacZ* transgenic mice with wild‐type *Trp53* (+/+) and *lacZ* transgenic mice hemizygous (+/−) or nullizygous (−/−) (12 weeks‐of‐age) were exposed to a single acute dose of 0 or 1 Gy ^56^Fe particles and then groups of animals were necropsied at 8 weeks postexposure for isolation of splenic T‐cells and measurement of mutant frequencies in the endogenous *hypoxanthine‐guanine phosphoribosyltransferase* (*Hprt*) gene using the T‐cell cloning assays as adopted from Walker et al. (1999).

^b^ Mann–Whitney *U* statistic test for difference between MF values of control versus irradiated mice of a given *Trp53* genotype.

^c^ To test for differences in radiation‐induced mutagenic effects between groups of *Trp53* wild‐type, hemizygous, and nullizygous mice, two‐way ANOVA and Tukey's post hoc multiple comparisons test were used.

The potential impact of *Trp53* status on the change in mutagenic response over time in female and male mice exposed to 0 or 1 Gy ^56^Fe particles at 10–13 weeks‐of‐age was evaluated by defining *Hprt* MFs in splenic T‐cells at 0, 2, 4, and 8 weeks postexposure (Table 2, Figure 1). At 2 weeks postexposure, *Hprt* MFs in irradiated *lacZ Trp53* ^+/+^, *Trp53* ^+/−^, and *Trp53* ^−/−^ mice for males and females combined were elevated significantly over respective sham‐irradiated control animal values measured at 0 weeks post‐exposure (respective *p* values = 0.05, 0.003, and 0.00005), with the mutagenic response in exposed *Trp53* ^−/−^ mice (22.1 ± 3.1 × 10^−6^) being significantly greater than in *Trp53* ^+/+^ mice (5.14 ± 3.1 × 10^−6^, *p* = 0.0004) and *Trp53* ^+/−^ mice (10.8 ± 6.1 × 10^−6^, *p* = 0.008). *Hprt* MFs were highest at 4 weeks post‐irradiation, with the maximum *Hprt* MFs being 11.5‐, 20.5‐, and 43.9‐fold higher than respective background levels in *Trp53* ^+/+^, *Trp53* ^+/−^, and *Trp53* ^−/−^ mice. Furthermore, *Hprt* MFs in irradiated *Trp53* ^−/−^ mice were 3.3‐ and 1.3‐fold higher than observed in *Trp53* ^+/+^ and *Trp53* ^+/−^ mice, respectively. Also, 4 weeks post‐irradiation, MFs in *Trp53* ^+/−^ mice were significantly elevated compared to exposed *Trp53* ^+/+^ mice (*p* = 0.0009). Last, the mutagenic responses in *Trp53* ^+/+^, *Trp53* ^+/−^, *Trp53* ^−/−^ mice necropsied at 8 weeks after exposure to 1 Gy ^56^Fe particles at 10–13 weeks‐of‐age (Table 2) approximated those in corresponding groups of mice necropsied at 8 weeks after irradiation at 12 weeks‐of‐age (Table 1), suggesting that this age difference at treatment was not a significant factor.

**TABLE 2:** *Hprt* mutant frequencies in T lymphocytes from spleens following acute iron particle irradiation of female and male *lacZ* transgenic mice with different *Trp53* genetic status^a^.

Weeks postexposure	Dose (Gy)	*Trp53* status	Sex	Observed MF (× 10^−6^)	*p*
0^b^	0	+/+	F	1.01 1.67	
			M	1.38^c^	
			F and M	1.35 ± 0.33	
	0	+/−	F	1.78 1.19	
			M	2.00^c^	
			F and M	1.66 ± 0.42	
	0	−/−	F	1.23 2.23	
			M	1.73^c^	
			F and M	1.73 ± 0.50	
2^b^	1	+/+	F	8.64^c^	
			M	2.83 3.95	
			F and M	5.14 ± 3.08	*p* = 0.05, irradiated vs. control +/+ mice^d^
	1	+/−	F	16.85 15.17 *x* = 16.0 ± 1.2	
			M	5.41 5.59 *x* = 5.5 ± 1.2	
			F and M	*x* = 10.8 ± 6.1	*p* = 0.003, irradiated female vs. male +/− mice^d^ *p* = 0.027, irradiated vs. control +/− mice^d^
	1	−/−	F	24.86 24.20 *x* = 24.5 ± 0.5	
			M	18.29 20.91 *x* = 19.6 ± 1.9	
			F and M	*x* = 22.1 ± 3.1	*p* = 0.034, irradiated female vs. male +/− mice^d^ *p* = 0.00005, irradiated vs. control −/− mice^d^
4	0	+/+	F	1.93 1.78	
			M	1.01 0.94	
			F and M	1.4 ± 0.5 (SD)	
	1	+/+	F	22.8 10.6	
			M	15.0 16.1	
			F and M	16.1 ± 5.0 (SD)	*p* = 0.0006, irradiated vs. control +/+ mice^d^
4	0	+/−	F	4.08 1.48	
			M	0.99 1.48	
			F and M	2.0 ± 1.4 (SD)	
	1	+/−	F	31.9 33.1	
			M	36.0 62.5	
			F and M	40.9 ± 14.5 (SD)	*p* = 0.003, irradiated vs. control +/− mice^d^ *p* = 0.0009, induced effect in +/− vs. +/+ mice^e^
4	0	−/−	F	1.70^c^ Dead on arrival	
			M	0.88 0.92	
			F and M	1.2 ± 0.5 (SD)	
	1	−/−	F	66.0 33.8	
			M	51.5 59.6	
			F and M	52.7 ± 13.9 (SD)	*p* = 0.0008, irradiated vs. control −/− mice^d^ *p* = 0.0013, induced effect in −/− vs. +/+ mice^e^ *p* = 0.14, induced effect in −/− vs. +/− mice^e^
8	0	+/+	F	1.17 1.56	
			M	1.16 1.29	
			F and M	1.3 ± 0.2 (SD)	
	1	+/+	F	2.41 2.46	
			M	6.92 4.39	
			F and M	4.0 ± 2.1 (SD)	*p* = 0.021, irradiated vs. control +/+ mice^d^
8	0	+/−	F	1.37 1.08	
			M	1.50 1.32	
			F and M	1.3 ± 0.2 (SD)	
	1	+/−	F	7.17 6.89	
			M	11.19 10.58	
			F and M	9.0 ± 2.2 (SD)	*p* = 0.0002, irradiated vs. control +/− mice^d^ *p* = 0.0096, induced effect in −/− vs. +/+ mice^e^
8	0	−/−	F	2.32 1.51 1.67	
			M	1.14 16.8^f^	
			F and M	1.7 ± 0.5 (SD)	
	1	−/−	F	17.9 12.3	
			M	26.9^c^	
			F and M	19.0 ± 7.4 (SD)	*p* = 0.002, irradiated vs. control −/− mice^d^ *p* = 0.005, induced effect in −/− vs. +/+ mice^e^ *p* = 0.023, induced effect in −/− vs. +/+ mice^e^

^a^ C57Bl/6 *lacZ* transgenic mice with wild‐type *Trp53* (+/+) and *lacZ* transgenic mice hemizygous (+/−) or nullizygous (−/−) (10–12 weeks old) were exposed to a single acute dose of 0 or 1 Gy ^56^Fe particles and then groups of animals were necropsied at 0, 2, 4, or 8 weeks post‐exposure for isolation of splenic lymphocytes and measurement of mutant frequencies (MFs) in the endogenous *hypoxanthine‐guanine phosphoribosyltransferase* (*Hprt*) gene using the T‐cell cloning assay adopted from Walker et al. (1999).

^b^ *Hprt* MFs in groups of mice receiving 1 Gy irradiation were compared to MFs in groups of control mice that were necropsied at time “0” or immediately before irradiation of the corresponding groups of irradiated mice that were held for two weeks before necropsy.

^c^ Mouse groups contained 2 or 3 animals of each sex except for the instances indicated by superscript “c.” Due to small sample sizes, data from both female and male mice in the same group were generally combined for statistical analysis with the few exceptions noted in another footnote.

^d^ Mann–Whitney U‐statistic was used to test for difference between MF values of control versus irradiated mice of a given *Trp53* genotype or to test for differences in MFs in irradiated female versus male mice.

^e^ To test for differences in radiation‐induced mutagenic effects between groups of *Trp53* wild‐type, hemizygous, and nullizygous mice, two‐way ANOVA and Tukey's post hoc multiple comparisons test were used.

^f^ Probable clonal expansion so that the MF value was not included in data for statistical analysis.

**FIGURE 1:**
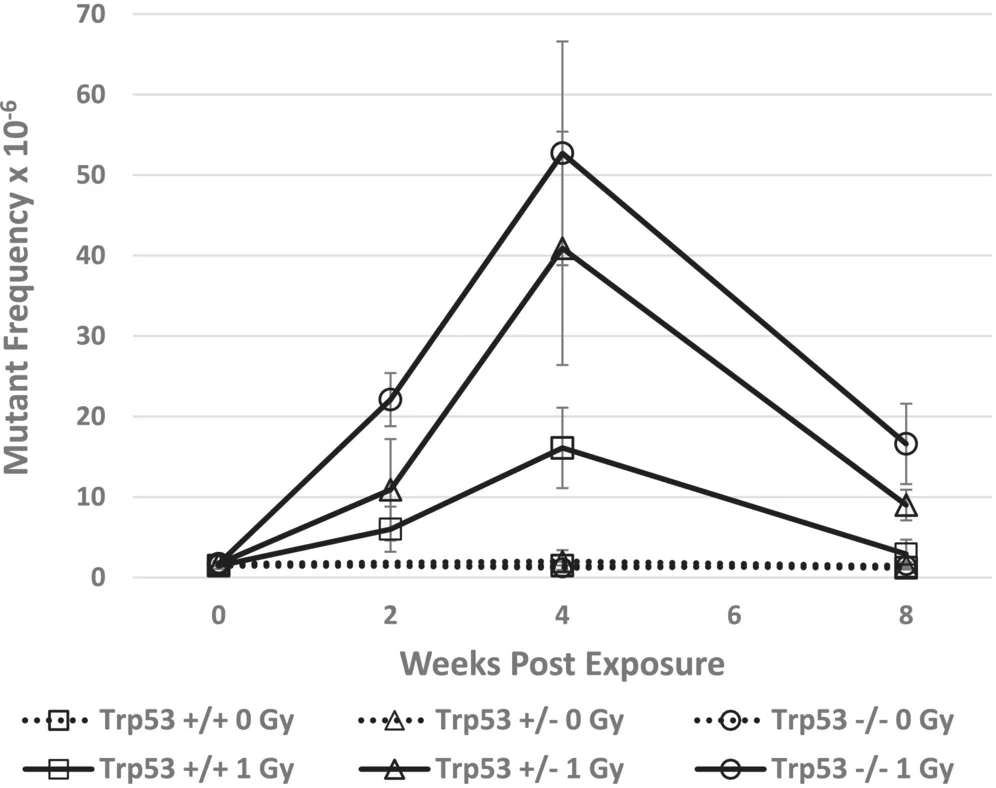
Relationship between time elapsed since acute exposure to 0 or 1 Gy ^56^Fe particles and the frequencies of *Hprt* mutant T‐cells isolated from spleen of *lacZ* mice with different *Trp53* genetic status. Mutant manifestation curves for control and irradiated *Trp53* ^+/+^ (wild‐type), *Trp53* ^+/−^ (hemizygous), and *Trp53* ^−/−^ (nullizygous) mice (10–12 weeks of age) were generated using MFs from individual female and male mice necropsied at 0, 2, 4, and 8 weeks after irradiation (*n* = 2/sex/*Trp53* genetic status/timepoint for respective control and irradiated groups; error bars = standard deviation).

Table 3 provides the estimated mutagenic potencies and Monte Carlo confidence intervals for the mutant manifestation curves for *Hprt* MFs in splenic T‐cells obtained from individual animals at 0, 2, 4, and 8 weeks postexposure of female and male *Trp53* ^+/+^, *Trp53* ^+/−^, *Trp53* ^−/−^ mice to 0 versus 1 Gy ^56^Fe particles (Figure 1). The estimated mutagenic potencies represent the number of mutant cells passing through the spleen over a given time period in treated versus respective control mice. The total induced effects over time following exposure to 1 Gy ^56^Fe particles were 3.0‐ and 5.3‐fold greater in *Trp53* ^+/−^ and *Trp53* ^−/−^ mice, respectively, compared to *Trp53* ^+/+^ mice.

**TABLE 3:** Estimated mutagenic potencies and Monte Carlo confidence intervals based upon *Hprt* mutant frequencies in T lymphocytes from spleens following acute 1 Gy high‐LET iron particle irradiation of female and male *lacZ* transgenic mice with different *Trp53* genetic status versus mutagenic potency estimates based upon *Hprt* mutant manifestation curves for splenic T‐cells from mice and/or rats exposed to one of several mutagenic chemicals including acrylonitrile, 1,3‐butadiene, and *N*‐ethyl‐*N*‐nitrosourea (ENU)^a^.

Species (age) and exposure	*Trp53* status	Mutagenic potency estimate	95% Confidence intervals	
			Upper	Lower
Mice, 1 Gy ^56^Fe^26+^ ions^b^	+/+	53	35	79
Mice, 1 Gy ^56^Fe^26+^ ions^b^	+/−	161	127	203
Mice, 1 Gy ^56^Fe^26+^ ions^b^	−/−	283	239	334
Rats (4 weeks old), 500 ppm acrylonitrile in drinking water for 4 weeks^c^	*Tp53* +/+ in rats	40.7	31.2	52.2
Rats (4–5 weeks old), 625 ppm butadiene by inhalation for 4 weeks^d^	*Tp53* +/+ in rats	7.2	4.2	10.5
Mice (4–5 weeks old), 625 ppm butadiene by inhalation for 4 weeks^d^	+/+	69.6	61.1	78.7
Mice (8–9 weeks old), 625 ppm butadiene by inhalation for 4 weeks^d^	+/+	57.0	44.7	71.5
Mice (22 days old), 40 mg ENU/kg bw given IP^e^	+/+	3800	3600	4000
Mice (8–9 weeks old), 40 mg ENU/kg bw given IP^e^	+/+	3700	3300	4000

^a^ Mutagenic potency of a given exposure a physical agent or chemical in mice/rats was estimated by subtracting the area under the curve of control animals from the area under the curve of exposed animals for each exposure scenario, as described in the Section 2 (Meng et al. 1998; Walker et al. 1999). Mutagenic potency is a relative value that represents the number of mutant cells passing through the spleen over the time‐period evaluated; that is, the mutagenic potency estimate is defined as [number of *Hprt* mutants/number of wild‐type cells] × [number of wild‐type cells passing through the spleen/unit of time].

^b^ C57Bl/6 *lacZ* transgenic mice with wild‐type *Trp53* (+/+) and *lacZ* transgenic mice hemizygous (+/−) or nullizygous (−/−) (10–12 weeks‐of‐age) were exposed to a single acute dose of 0 or 1 Gy iron ions and then groups of animals were necropsied at 0, 2, 4, or 8 weeks postexposure for isolation of splenic lymphocytes and measurement of *Hprt* mutant frequencies.

^c^ Groups of female F344 rats were necropsied at 0, 2, 4, 6, or 8 weeks postexposure to 0 or 500 ppm acrylonitrile in drinking water for 4 weeks (Walker et al. 2020).

^d^ Groups of female F344 rats or B6C3F1 mice were necropsied immediately before exposure and 0, 2, 4, 5, 6, or 8 weeks postexposure to 0 or 625 ppm 1,3‐butadiene by inhalation for 4 weeks (6 h/day, 5 days/week) (Meng et al. 2007).

^e^ Male B6C3F1 mice were given single intraperitoneal injections of 0 or 40 mg ENU/kg bw at 22 days (weanlings) or 8 weeks of age, and groups of animals were necropsied up to 70 days postexposure (Walker et al. 1999).

## 4 Discussion

The mutant manifestation curves and mutagenic potency estimates for *Hprt* MFs in splenic T‐cells (Figure 1, Table 3) demonstrate that while 1 Gy ^56^Fe particles caused significant increases in mutations in *Trp53* ^+/+^ mice, *Trp53* ^+/−^ mice were more sensitive, and *Trp53* ^−/−^ mice were the most sensitive to radiation‐induced mutagenesis. The shapes of the mutant manifestation curves, with a rise and fall in *Hprt* MFs in spleen over time in irradiated mice, reflect the induction of mutations primarily in pre‐thymocytes in bone marrow and the trafficking of mutant T‐cells through the thymus to the spleen and other lymphoid organs (Walker et al. 1999; Judice et al. 2023).

The potential impact of *Trp53* gene status on genotoxic susceptibility is further illustrated in Table 3 by differences in the mutagenic potency estimates for *Trp53* ^+/+^, *Trp53* ^+/−^, and *Trp53* ^−/−^ mice exposed acutely to 1 Gy ^56^Fe particles compared to earlier mutagenic potency estimates in mice and/or rats receiving a single exposure to the highly potent mutagen, *N*‐ethyl‐*N*‐nitrosourea, or multiple exposures to environmental/industrial chemicals, acrylonitrile or 1,3‐butadiene, which are classified as known human carcinogens by the International Agency for Research on Cancer. Overall, these findings show that defining the manifestation of reporter gene mutations in T‐cells isolated from spleens of irradiated mice can be a useful model for investigating haploinsufficiency, pathogenic polymorphisms, and null mutations in *Trp53* and other genes (*BRCA1*, *ATM*) known to increase sensitivity to ionizing radiation (Su et al. 2010).

Several earlier studies investigated the genotoxic effects of ^56^Fe particles in transgenic mice by measuring the frequencies of *lacZ* mutations in spleen and frequencies of micronucleated reticulocytes (MN‐RETs) as a biomarker for clastogenic agents that cause DNA double‐strand breaks (Dertinger et al. 2007). In *Trp53* ^+/+^ mice at 8 weeks postexposure to 1 Gy ^56^Fe particles, *lacZ* MFs in spleen were increased by 2.6‐fold above background (Chang et al. 2001a) compared to an ~11‐fold increase in *Hprt* MFs above background in splenic T‐cells at the same timepoint post‐irradiation in the current study (Tables 1 and 2). Radiation‐induced alterations in the spectrum of *lacZ* mutations were driven in part by changes in the frequency of mutants with deletions extending past the transgene into mouse genomic DNA (Chang et al. 2001a). At 72 h postexposure to 0 or 1 Gy iron particles, MN‐RETs were increased by 2.3‐, 2.5‐, and 4.3‐fold in *Trp53* ^+/+^, *Trp53* ^+/−^, and *Trp53* ^−/−^ mice versus respective controls (Chang et al. 2000).

Direct comparisons of data presented here to earlier evaluations of mutations induced by ^56^Fe particles in spleen are complicated by whether MFs were measured in a timely fashion in transgenes of whole spleen or in endogenous reporter genes of splenic T‐cells. The problem with such comparisons is that MFs measured in T‐cells isolated from spleen after mutagen exposure are dependent upon the trafficking of mutant cells from bone marrow through the thymus to spleen, while analysis of whole spleen for induced mutations reflects mutagenesis also occurring within the parenchyma of a complex secondary immune organ (Cesta 2006).

The importance of suitable sampling times in experimental designs for evaluating radiation‐induced mutagenic effects in splenic T‐cells is illustrated by comparisons of *Hprt* MF data in female versus male mice in Table 2 and comparisons to *Aprt* MFs in irradiated mice and to *Hprt* MFs in rodents exposed to chemical mutagens (Table 3). First, while MF data from comparable groups of female and male mice in the current study were combined due to the small sample size at each timepoint, scrutiny of data in Table 2 suggests that the trafficking of *Hprt* mutant T‐cells from bone marrow to spleen occurred at a ~1‐week faster rate in irradiated females compared to males. Second, while there was no significant increase in *Aprt* MFs in T‐cells isolated from spleens 3–5 months postexposure of mice to 0.5 or 2 Gy ^56^Fe particles, there was a significant shift in mutational spectra related to the persistence of a subset of mutant T‐cells that remained after trafficking to the spleen (Grygoryev et al. 2016). The mutant manifestation curves in Figure 1 confirm the authors' conclusion that MFs in endogenous reporter genes of T‐cells isolated from spleen should be defined as a function of time after irradiation of rodents. Nevertheless, the finding that the maximum *Hprt* MFs in spleen occurred at ~4 weeks after exposure of 10–13 week‐old mice to 1 Gy ^56^Fe particles was unexpected. Earlier studies of rodents exposed to chemical mutagens found that the time required to reach peak *Hprt* MFs in spleen approached the age of the mouse/rat at the time of a single exposure or the last of multiple exposures (Walker et al. 1999; Meng et al. 2007). A potential explanation for this difference is that radiation causes DNA double‐strand breaks that can induce medium‐to‐large deletion mutations in the *Hprt* and adjacent genes (Rothkamm et al. 2008), yielding autoreactive T‐cells that are eliminated via negative selection in the thymus. The net effect is that T‐cell trafficking from thymus to spleen after irradiation is different from that found after induction of point mutations by chemical mutagens in rodents.

Future efforts to characterize the effects of high‐ and low‐LET irradiation on mutagenic potency, mutagenic specificity, and/or patterns in persistence of mutated erythroid and lymphoid cells in bone marrow should be enhanced by the recent advancement of modern flow cytometry methods for rapid, sensitive measurements of MFs in the X‐linked *phosphatidylinositol glycan anchor biosynthesis class A* (*Pig‐a*) reporter gene of erythrocytes (RBCs) and T‐cells (Dobrovolsky et al. 2019; Dertinger et al. 2021). Ohtani et al. (2014) defined the change in MF over time and persistence of *Pig‐a* mutations in peripheral blood RBCs from *Trp53* ^+/+^ or *Trp53* ^−/−^ mice exposed to 0 or 1 Gy x‐rays. Their results suggest that the persistence of *Pig‐a* mutant RBCs after x‐rays is affected by p53 function, distinct types of mutations impacting erythrophagocytosis, and RBC lifespan. For instance, *Pig‐a* MFs reached a peak at 28 days post‐irradiation in both *Trp53* ^+/+^ or *Trp53* ^−/−^ mice followed by decreases in MFs associated with continuous replacement of RBCs over the ~47‐day lifespan of mouse erythrocytes (Ohtani et al. 2014). Parallel studies in splenic T‐cells can be accomplished using the general approach applied in the current report by measuring the manifestation of mutations at the *Pig‐a* locus in mice/rats exposed to alpha particles, protons, neutrons, γ‐rays, or x‐rays. Crucially, concurrent measurements of the change over time in the frequencies of *Pig‐a* MFs in splenic T‐cells and peripheral RBCs, as well as MN‐RETs, offer a powerful tool to distinguish agents that offer good protection of the mouse/rat hematopoietic system for investigating/repurposing drugs to prevent or counter injuries from acute or delayed effects of radiation exposure (reviewed in Singh and Seed 2021).

## Author Contributions

L.L.‐M. and V.E.W. designed the study. L.L.‐M. arranged crucial radiation exposures for the project. V.E.W., D.M.W., and V.N.D. performed the experimental work. V.E.W. and D.M.W. collated, curated, and analyzed the data; prepared the tables and figure; and wrote the draft manuscript. V.N.D. revised the manuscript critically for important intellectual content. All authors provided intellectual input and approved the final version of the manuscript.

## Funding

This study was supported by NASA Grant #NAG5‐6177 and Eunice Kennedy Shriver National Institute of Child Health and Human Development Grant #R01 HD33648.

## Disclosure

This manuscript reflects the views of the authors and does not necessarily reflect those of the U.S. Food and Drug Administration. Any mention of commercial products is for clarification only and is not intended as approval, endorsement, or recommendation.

## Data Availability

The data that support the findings of this study are available from the corresponding author upon reasonable request.
